# Relationship between Psychological Pain and Social Cognition with the risk of suicidal behavior in depressed patients in remission: a pilot study

**DOI:** 10.1192/j.eurpsy.2025.2350

**Published:** 2025-08-26

**Authors:** X. Bros, I. Parra, M. T. Muñoz, B. Soley, M. Perez, D. Palao

**Affiliations:** 1 Psychiatry, Universitat Autònoma de Barcelona, Barcelona; 2 Psychiatry, Taulí Hospital, Sabadell; 3 CIBERSAM, Barcelona; 4 I3PT, Sabadell, Spain

## Abstract

**Introduction:**

Suicide is a major public health problem. Psychological Pain (Psychache) and Social Cognition (SC) may have potential clinical significance. The aim of this study is to determine whether they are clinically relevant in patients with a history of suicide attempts and Major Depression (MD) in remission.

**Objectives:**

1. To investigate the severity of Psychache in patients with MD who have attempted suicide.
2. To identify changes in SC associated with an increased risk of suicidal behavior.
3. To identify clinical subgroups of patients according to the SC and Psychache typology.

**Methods:**

A controlled cross-sectional observational study is being conducted comparing two groups assessed with a clinical diagnostic interview and a psychological assessment including measures of SC and Psychache: 1) 60 patients with a history of suicide attempts (more than 6 months prior to the study), diagnosed with MD (DSM-5) at the time of their last attempt, and in remission when evaluated (HRDS<15). 2) 60 age/gender matched healthy case controls.

**Results:**

Preliminary results from a group of 23 patients matched with 23 healthy controls:

1. Psychache: a) Patients vs. Controls: Patients, despite being in remission of depression, have a higher level of Psychache than controls (p<0.001). b) Patients: The level of current Psychache is significantly lower than at the time of the suicide attempt (p<0.001). c) The level of current Psychache correlates significantly with the depression severity (HDRS) (r=0.77). This correlation is significant in the patient group (p<0.001) but not in the control group. d) The results obtained on the Total Psychache Scale do not differ from those obtained on the Unbearable Psychache subscale.
2. Social Cognition: Overall scores on two of the Social Cognition measures (RMET and Hitting Task) were not significantly different between the patient and control groups. In the case of the MASC test, the global scores of the group of patients (with a history of suicide attempts) are significantly lower than those of the control group (p<0.05).

Table 1

**Table:** 

	CONTROLS	PATIENTS	P-value
Psychache Total	Mean14.7 SD (2.88)	31.3 (14.0)	<0.001
Subtest Unbearable	3.0 (0)	6.3 (3.70)	<0.001
RME (SC)	26.0 (3.02)	23.8 (3.76)	0.062
Hitting (SC)	8.7 (1.14)	8.6 (0.99)	0.424
MASC Total (SC)	31.1 (3.92)	28.3 (4.78)	<0.05

**Conclusions:**

a) Patients with stable depression and a history of suicide risk maintain a significant level of Psychache, which is higher than in the control group. b) The use of the Unbearable Psychache subscale -with only 3 items- discriminate clearly between the patient (suicide attempters) and the control groups: its use in primary care should be considered. c) Differences between patients and controls in SC were not significant in two of the three scales used, but were significant in one (MASC). This should be confirmed and analyzed in the full sample.

**Disclosure of Interest:**

None Declared

